# Outcomes and Surgical Complications in Kidney Transplantation

**Published:** 2017-05-01

**Authors:** F. Reyna-Sepúlveda, A. Ponce-Escobedo, A. Guevara-Charles, M. Escobedo-Villarreal, E. Pérez-Rodríguez, G. Muñoz-Maldonado, M. Hernández-Guedea

**Affiliations:** University Hospital "Dr. José Eleuterio González", Autonomous University of Nuevo León, Monterrey, México.

**Keywords:** Kidney transplantation, Intraoperative complications, Graft survival, Kidney failure, chronic, Morbidity

## Abstract

**Background:**

Kidney transplantation is the most cost-effective therapy for end-stage renal disease. Post-operative complications account for 15%–17% of all cases and are associated with significant morbidity. Currently 4.8% of post-transplantation patients have returned to dialysis. Our center’s main transplant origin is cadaveric donation.

**Objective:**

To review surgical complications of kidney transplantation over the past 5 years.

**Methods:**

This was an observational descriptive study that included all patients from 2011 to 2015.

**Results:**

A total of 55 cases were reviewed. Diabetic nephropathy was the etiology in 30.9% of cases. Post-surgical complications occurred in 12.7% of patients with a post-operative mortality of 4%. Graft survival at 1 year was 82.4% with a 91% 1-year patient survival.

**Conclusion:**

Early identification and treatment of surgical complications are critical for patient and graft survival. Complications are low but significant.

## INTRODUCTION

Kidney transplantation is considered the most cost-effective therapy for end-stage renal disease (ESRD). Since the first human kidney transplantation by Joseph Murray in 1954 [1], improvements in morbidity and mortality have been attributed to patient selection, advances in surgical technique, peri-operative management, and immunosuppressive regimens.

Complications of renal transplantation can be classified as pathological or surgical. Pathological complications include rejection, infection, and cardiovascular events, while surgical complications involve vascular and urological complications, lymphocele, wound infection, and herniation.

Despite all advances, graft-endangering complications are primarily of vascular etiology. Vascular complications account for 3%–15% of all cases [2]. These include thrombosis or stenosis of the renal artery or vein. Other rare complications are the formation of aneurysms, arteriovenous fistulas or hematomas, which can be diagnosed and treated by interventional radiology. Some risk factors include poor surgical technique, torsion or compression of vessels, the presence of multiple renal vessels or a renal artery atheroma. Other factors involved are the presence of anatomical variations, such as double ureters and multiple renal arteries or veins, which represent a challenge for the transplant surgeon. The most common variant, which is found in 8%–30% of all potential kidney donors, is the presence of multiple renal arteries [3].

According to the United Network of Organ Sharing (UNOS), graft survival at two years is 90%; however, in 5-year surveillance reports, 30% of patients have lost the graft or died with a functioning kidney [4]. The main causes of early graft failure (first six months) are acute rejection, technical problems, and a nonviable kidney [5]. In the case of chronic failure, it is caused by death because of a non-kidney-related problem with a functional graft and chronic kidney disease. Currently 4.8% of post-transplantation patients have returned to dialysis [6].

Knowledge of the incidence of clinical manifestations and management of surgical complications is necessary for all kidney transplant surgeons. The objective of this study was to report our experience with the epidemiology, peri-operative variables, and surgical complications of kidney transplantations performed over the past five years in our center.

## MATERIALS AND METHODS

An observational descriptive study was conducted between 2011 and 2015 at “Dr. José Eleuterio González” University Hospital. All renal transplant recipients were included. Demographic variables, medical history, laboratory tests, details of the surgical procedure and post-transplantation follow-up at three months and one year were reviewed.

Patients with a complete transplantation protocol, including cardiovascular, infectious, psychosocial and urological evaluations were included. All patients underwent panel reactive antibodies (PRA), human leukocyte antigens (HLA), and cross-lymphocyte cytotoxicity tests. All patients signed informed consent and all organ donations were cadaveric.

Regarding the surgical technique, after washing the bladder with amikacin and placement of a Foley catheter, we proceeded to place the donated kidney in the right or left iliac fossa using side-to-end anastomosis of the renal artery and vein to the iliac vessels. To perform neoureterostomy we used the antireflux Laedbetter-Politano and the Lich-Gregoir techniques according to the decision of the operating surgeon. Statistical analysis was performed using SPSS v20 (SPSS, Inc., Chicago, Ill).

## RESULTS

A total of 55 heterotopic cadaveric kidney transplantations were performed in 31 (56%) men and 24 (43%) women. The mean age of recipients was 49.5 years; the mean body mass index (BMI) was 27 kg/m^2^. The origin of the nephropathy was type 2 diabetes mellitus in 17 (31%); hypertension in 10 (18%); and other pathologies such as nephrolithiasis, glomerular diseases, or idiopathic in the remaining 28 patients. Of the total patients, 48 were on renal replacement therapy for a mean period of 2.18 years with 21 (43%) patients in hemodialysis and 27 (57%) in peritoneal dialysis (Table 1).

**Table 1:** Sociodemographic factors in kidney transplant recipients (n=55)

Variable	n (%)
Sex	
Male	31 (56)
Female	24 (43)
Mean body mass index (kg/m^2^)	27
Nephropathy	
Type 2 diabetes mellitus	17 (31)
Arterial hypertension	10 (18)
Other	28 (51)
Kidney replacement therapy	
Mean time (yrs)	2.18
Hemodialysis	21 (43)
Peritoneal dialysis	27 (57)
Surgical procedure	
Mean cold ischemic time (hrs)	8.4
Mean surgical time (hrs)	4.15
Mean trans-operative bleeding (mL)	450
Multiple renal arteries	9 (16)

From the transplanted kidneys, 49 (89%) were located in the right iliac fossa and 6 (11%) in the left. Out of the donated specimens, 33 (60%) were right kidneys and 22 (40%) were left.

The mean time of cold ischemia was 8.44 hours; it was 4.15 hours for the surgical procedure and 450 mL for approximate total blood loss. Nine (16%) patients had multiple renal arteries (MRA). In the immediate post-operative period, only one patient received more than three units of packed red cells and four patients remained in critical care for more than 48 hours. The mean hospital stay was 12 days. For ureterovesical anastomosis, we performed 47 (85%) Laedbetter-Politano and 7 (12%) Lich-Gregoir procedures. Vascular anastomoses were performed side-to-end and in one case with three renal arteries they were anastomosed together with a cadaveric graft iliac vein.

There were 7 (13%) post-surgical and 5 (9%) chronic complications, stratified by etiology (Table 2). Two (4%) deaths were reported from nosocomial pneumonia and pulmonary embolism, and two (4%) deaths in the subsequent three months because of sepsis. There was only one patient with graft loss that could be attributed to a surgical complication related to a ureteral injury with ferula placement and posterior urinoma formation.

**Table 2:** Surgical complications of kidney transplant recipients (n=55)

Parameter	n (%)
Post-operative	7 (13)
Hematoma	2 (4)
Wound Infection	2 (4)
Ureteral injury	1 (2)
Arterial injury	1 (2)
Venous thrombosis	1 (2)
Chronic	5 (9)
Incisional hernia	2 (4)
Arterial stenosis	1 (2)
Vesicoureteral occlusion	1 (2)
Lymphocele	1 (2)

Pre-operative, post-operative and 3-month follow-up lab data were reviewed (Table 3). For statistical analysis, we compared the groups with and without complications, and found differences in only pre-operative creatinine and albumin, in addition to the delay in organ function statistically significant (p<0.05).

**Table 3:** Pre-operative, post-operative and follow-up lab test results of uncomplicated patients

Value	Pre-operative	Post-operative	3-month follow-up
Blood urea nitrogen (BUN)	59.9	34.1	22.5
Creatinine	9.6	1.7	1.3
Hemoglobin	11.1	9.4	11.6
Albumin	3.7	2.8	4.2
Tacrolimus blood level	–	12.8	10.6

## DISCUSSION

Most of our patients were overweight with an average BMI of 27 kg/m^2^, which represented an extra challenge in surgical procedures. In our surgical technique we commonly transplant on the right side of the patient, transplant was done in the left side in the case of a previous transplant or surgical history. 

Our prevalence of 16% of multiple renal arteries represented a surgical challenge. We performed multiple anastomoses or used iliac vein graft to gain additional working space. In the case of different techniques used for the vesicoureteral anastomosis this was chosen by the surgeon according to their experience.

**Figure 1:**
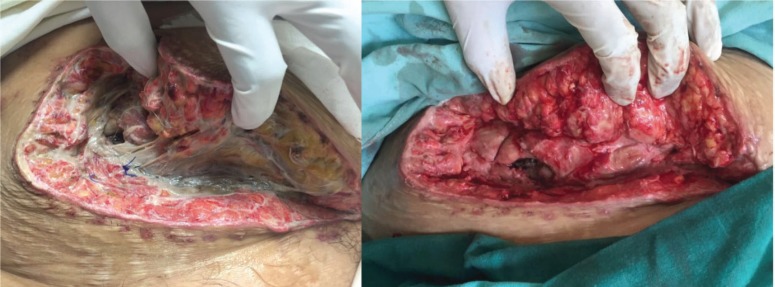
Infected surgical wound before (left) and after (right) vacuum-assisted closure

In regards to our surgical complications, the hematoma formation was related to a biopsy and in both cases open surgery was the preferred approach. Wound infection was treated with a vacuum device without complications (Fig. 1). The ureteral injury was a small puncture during dissection, which was primarily repaired and a ferula was placed. The arterial injury was related to an injury of a polar artery that was ligated. In the case of venous thrombosis surgical management was preferred. 

Chronic complications include incisional hernia, which was repaired with mesh at six months. The arterial stenosis was managed with endoluminal stent placement. The case of vesicourethral occlusion was treated with ferulization of the anastomosis. Conservative treatment was performed in our case of lymphocele.

Laboratory results include the expected decrease in blood urea nitrogen and creatinine with a significant increase in albumin over the following three months. Tacrolimus blood levels remained stable during the follow-up. The only statistically significant variables by comparing complicated to uncomplicated patients were creatinine, albumin, and delayed graft function. Low albumin and elevated creatinine in these groups were associated with delayed tissue repair.

Surgical complications in kidney transplantation are usually associated with reoperation and can rapidly and severely affect graft survival. To minimize the morbidity and mortality, we must quickly diagnose and treat appropriately the problem. Surgical complications rarely lead to graft loss, with the exception of vascular pathology [7]. In our study we reported an incidence of 12.7% of surgical complications similar to the 15.9% reported in the literature [8].

Renal arterial stenosis is the most common vascular complication; it occurs in 3%–23% of all transplantations in the first 12 months [9, 10]. This rate is associated with the end-to-end anastomosis and cadaveric donor grafts. If left untreated, this pathology would lead to kidney dysfunction, resistant hypertension, and subsequent deterioration of the graft [11]. The main management is endoluminal percutaneous angioplasty with or without stenting and the main markers of recovery are improved renal function and blood pressure. 

The incidence of arterial thrombosis is 0.3%–6.1% [12]. It is most common in the first two weeks of transplantation, 80% in the first month and 93% in the first year [13]. After the first month, thrombosis of the renal artery occurs mainly because of rejection or a high degree of stenosis. 

In the case of renal vein thrombosis, it is due to the spread of deep vein thrombosis from the lower extremity or extrinsic compression by a collection. It is characterized by the presence of oliguria, hematuria, elevated creatinine and pain at the surgical site [14]. Thrombosis is diagnosed with Doppler ultrasound and treated surgically by laparotomy, thrombectomy, or even graft nephrectomy. There are multiple reports for endoluminal management of thrombosis. However, the role of interventional radiology is not well defined [15].

The renal vein thrombosis is a dramatic early vascular complication of renal transplantation, with a reported prevalence between 0.5% and 4%. Despite its low incidence, it is one of the most important causes of graft loss in the first month after transplantation [16]. 

Hematoma formation is a frequent minor vascular complication that occurs in the post-operative period. The most common source is a small leakage of the vascular anastomosis or minor bleeding from the renal surface or the surrounding tissues. When hematomas grow and produce clinical signs or symptoms by pressure, it may lead to graft dysfunction and thrombotic complications. Ultrasound or computed tomography (CT) are used for diagnosis [17]. Hematoma may be complicated with infection that can be treated with ultrasound-guided percutaneous drainage. Large hematomas in the immediate post-operative associated with hypovolemic shock should be treated as a surgical emergency.

Urological complications are the most common complications in the late period after kidney transplantation, presenting an incidence ranging from 2.5% to 12.5% [18], lower than that occurred in the beginning of renal transplantation era of 25% [19]. These complications are a major cause of morbidity, delayed graft function, and increased hospitalization costs. Ischemia of the donor ureter and failure in surgical technique are the leading causes of urological complications [20]. 

High immunosuppressing regimens, acute rejection, BK virus, and infection also lead to obstruction. A low-steroid protocol and meticulous surgical technique decreases the incidence [21] and the use of old graft donors increases it. Different techniques for the ureterovesical anastomosis have been described, however they do not affect the incidence of complications [22]. Stenosis and fistula are the main causes and managed with open surgery.

In the case of multiple renal arteries (MRA), many centers prefer not to use these kidneys when other suboptimal conditions such as advanced donor age, coexist [23]. The main concerns are the technical complications associated with anastomosis and graft loss. More than 70% of the kidneys have a single renal artery [24], which normally divides in the hilum. Sometimes, it can bifurcate early or near the origin of the aorta and the only option to take the kidney is to divide the segmental arteries and perform a challenging anastomosis when they are short. The increased difficulty and more time needed for these anastomoses also increased the ischemia time causing a negative impact on the rate of acute tubular necrosis (NTA) and graft survival [25].

Sometimes, a small low polar renal artery may be the only irrigation the ureter has, so these vessels cannot be sacrificed during implantation [26]. If this vessel is occluded by a thrombus, the ureter will be ischemic and ultimately necrotic [27]. In these cases, when MRA are present, complications such as stenosis and thrombosis are more likely to occur [28]. However, it does not adversely affect patient or graft survival [29].

Lymphoceles occur during the dissection process by opening the lymphatics. In most patients, these fluid collections are asymptomatic and are found in ultrasound examination not requiring any invasive treatments. Larger collections may be associated with dilation of the collector system, pain, fever, and declined renal function. In these cases, ultrasound-guided aspiration should be done. Some cases may require insertion of a nephrostomy tube, although we can also open a non-infected lymphocele to peritoneal cavity. Wound infection rate in these patients is high due to the immunosuppression, but it may be managed with antibiotics. Hernia formation requires surgical repair.

The four deaths we reported were in patients older than 60 years, with type 2 diabetes mellitus, hypertension, and with renal replacement therapy. One mortality was directly associated with graft failure at three months because of a ureteral injury with posterior urinoma formation. We were not able to establish independent prognostic factors of statistical significance for surgical complications based on complications analyzed.

It is recommended that routine and emergency examinations with Doppler ultrasound are done for graft preservation. Surgical complications of kidney transplantation can be minimized with standardization of surgical techniques. Additionally, each organ should be used in its best condition. Early identification and treatment of these complications are critical to patient and graft survival. Thanks to advanced surgical techniques, kidney transplantation continues to be a safe treatment modality.
